# Recruiting Human Microbiome Shotgun Data to Site-Specific Reference Genomes

**DOI:** 10.1371/journal.pone.0084963

**Published:** 2014-01-15

**Authors:** Gary Xie, Chien-Chi Lo, Matthew Scholz, Patrick S. G. Chain

**Affiliations:** 1 Genome Science Group, Los Alamos National Laboratory, Los Alamos, New Mexico, United States of America; 2 Microbial and Metagenome Program, Joint Genome Institute, Walnut Creek, California, United States of America; The Roslin Institute, University of Edinburgh, United Kingdom

## Abstract

The human body consists of innumerable multifaceted environments that predispose colonization by a number of distinct microbial communities, which play fundamental roles in human health and disease. In addition to community surveys and shotgun metagenomes that seek to explore the composition and diversity of these microbiomes, there are significant efforts to sequence reference microbial genomes from many body sites of healthy adults. To illustrate the utility of reference genomes when studying more complex metagenomes, we present a reference-based analysis of sequence reads generated from 55 shotgun metagenomes, selected from 5 major body sites, including 16 sub-sites. Interestingly, between 13% and 92% (62.3% average) of these shotgun reads were aligned to a then-complete list of 2780 reference genomes, including 1583 references for the human microbiome. However, no reference genome was universally found in all body sites. For any given metagenome, the body site-specific reference genomes, derived from the same body site as the sample, accounted for an average of 58.8% of the mapped reads. While different body sites did differ in abundant genera, proximal or symmetrical body sites were found to be most similar to one another. The extent of variation observed, both between individuals sampled within the same microenvironment, or at the same site within the same individual over time, calls into question comparative studies across individuals even if sampled at the same body site. This study illustrates the high utility of reference genomes and the need for further site-specific reference microbial genome sequencing, even within the already well-sampled human microbiome.

## Introduction

Current data place the number of bacteria living in or on the human body as outnumbering the total number of human cells by a factor of 10 to 1 [1], [2]. Past developments in the field of human microbiome research has been summarized in a series of papers (e.g. [3], [4], [5], [6], [7], [8], [9], [10], [11], [12], [13]). While most of these studies have relied solely on community 16S rRNA gene amplicon analyses (which suffer the risk of PCR-induced biases), two recent companion papers published by the Human Microbiome Project Consortium (HMPC) describe both 16S rRNA as well as random shotgun sequencing data of healthy human subjects sampled from 5 major body sites and up to 18 sub-sites [14], [15]. This large effort used the shotgun metagenomic data to assess functional profiles and to investigate taxonomic classification. The latter analysis was performed using a reference database of 649 non-redundantly selected genomes for read-mapping and for comparisons of resulting alpha and beta diversity with 16S results. More specific species-level assignments were performed using either select pathogen genomes or marker genes [15].

Here, we made use of all available draft and completed genomes (at time of study), which comprises 1583 human-associated reference genomes, including 720 HMPC reference strains (isolated from the same 5 major body sites), as well as 1197 reference genomes from organisms not associated with the human body. This broader reference database allows us to explore the relative contribution of each reference, and the correlations of metagenome read recruitment with particular reference genomes from each body site. We used a subset of available HMPC metagenomic data to explore both spatial and temporal microbial diversity within specific individuals as well as differences among individuals sampled at the 5 major body sites. Given that nearly all metagenome data from some body site samples can be mapped to available reference genomes while other samples show very poor read-recruitment, we explored the value of having site-specific references (i.e. isolated from the same site as the metagenome sample). Additional genomes of organisms isolated from the same environment as that under study does enable broader analysis and interpretation of metagenomic data.

## Methods

### Metagenomic sequences dataset

The library construction, sequencing, and screening for human DNA contamination have been described in the standard operation procedures (http://www.hmpdacc.org). A total of 55 of the publicly available HMP metagenomic samples recovered from 5 human body sites (oral, skin, airway, gastrointestinal tract, and urogenital tract/vagina) along with associated metadata were selected based on 5 per oral sub site and 2 per other site and downloaded from HMP DACC site (http://www.hmpdacc.org). The number of Illumina reads and total bp from each sample used in this study are shown in Table S1 in File S2, along with the NCBI Sequence Read Archive numbers. Briefly, the samples used include SRS013946, SRS014473, SRS014687, SRS015060, SRS019025, SRS011090, SRS011144, SRS011247, SRS011310, SRS012281, SRS062878, SRS013947, SRS014474, SRS015061, SRS019026, SRS019126, SRS013942, SRS014468, SRS014692, SRS015055, SRS019120, SRS013950, SRS014107, SRS014477, SRS014691, SRS015064, SRS011098, SRS011126, SRS011152, SRS011255, SRS011343, SRS013948, SRS014475, SRS014689, SRS015062, SRS019027, SRS011086, SRS011115, SRS011140, SRS011243, SRS011306, SRS011105. A schematic of the body sites sampled for this HMP study was produced by Sitepainter visualization tool from Knight, Perrung, and Gonzalez, University of Colorado (http://www.hmpdacc/sp) and further modified from [16].

### Reference genome dataset

All reference genomes downloaded from JGI-IMG site (http://www.hmpdacc-resources.org/cgi-bin/imgm hmp/main.cgi) along with associated meta-data. This dataset includes 1358 complete and 1422 draft genomes. Genomes were classified by location of isolation (human/non-human) and specific body sites (oral, skin, airway, gastrointestinal tract, and urogenital tract/vagina). To assist with downstream identifications, all reference genomes were tagged with a unique IMG-taxa id. This allows a read mapping hit to any genome to be easily traced back to its parent taxonomic name, and was a required step to enable the creation of abundance metrics at each taxonomic level. The final reference database that was used in the analysis for this paper contained 2780 bacterial genomes.

In addition, we divided the 2780 bacterial reference genomes into three categories based on their corresponding metadata: 1) those isolated from a specific body site to enable comparisons with metagenomes isolated from the same body site (e.g. oral, GI, UI, skin, airway, et al); 2) those affiliated with other human body sites (or of known human origin but with no assigned body site); 3) those not affiliated with the human body (or not described to be of human origin). For the top ten reference genome found from each of these three categories, the host and body site metadata fields were manually curated.

### Metagenome sequence mapping

**Figure 1:**
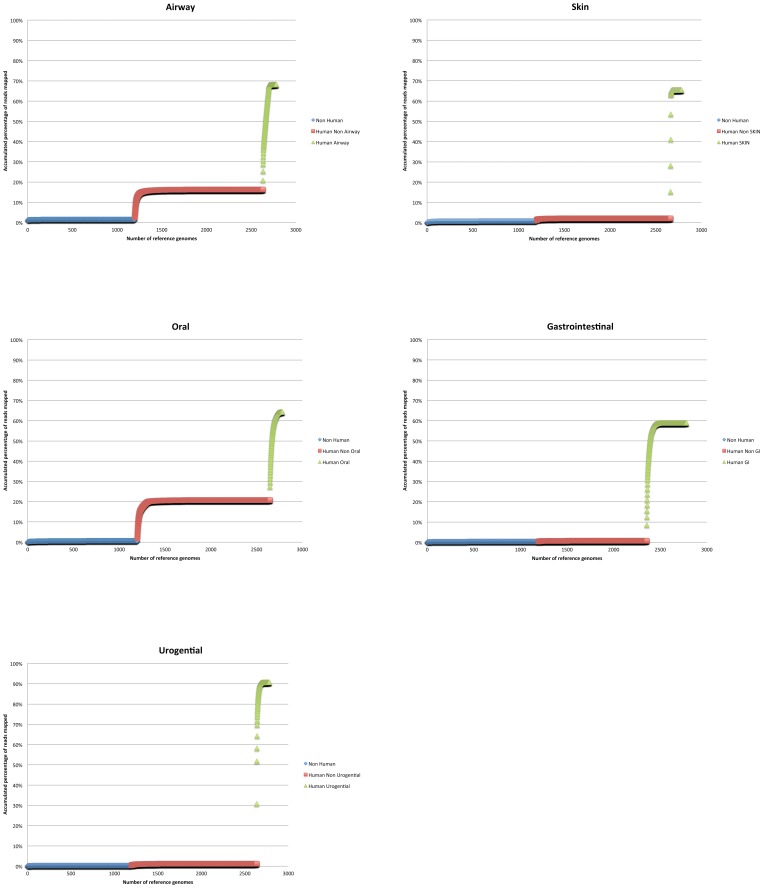
Proportion of reads mapped to 2780 reference genomes grouped into three categories of references. Reference genomes found in the same body site as the metagenome sample are in Green, those found and isolated from a different body site are in Red, and those references that are not known to be affiliated with humans are in Blue. The percentage of all reads mapped is shown along the y-axis in an accumulation curve. The five major body sites are shown with the reference genomes are along the x-axis in decreasing read-mapping abundance for each of the three categories of references.

Processed Illumina reads were mapped onto reference genomes in order to classify and calculate organism abundances. The final set was aligned using the CLC aligner (CLC Assembly Cell package, CLC bio - http://www.clcbio.com/) with the following parameters: -length fraction 0.75 -similarity 0.8 -p fb ss 180 250. The read-mapping cutoff (>80% identity) is based on the global alignment percent identity (defined as the number of identities between read and reference, divided by the length of the read); thus requiring 80% identity over 80% length of the read. In the event of multiple hits with equivalent scores, a random site is selected. In order to avoid misinterpreting spurious matches to genomes, the top genera must have at least 1% genome coverage (shown in Table S1 in File S2 and used for all downstream analysis). Both the paired end set and the fragment file were aligned in a single execution of the software. Rarefaction-like curves were calculated by parsing the contig.info file using custom perl scripts. Within each of the three categories, the reference genomes were sorted in descending order with respect to the number of reads mapped. Accumulation curves for the percentage of all reads mapped to all these sorted references were plotted after merging all data per 5 major body sites (Figure 1 and Figure S1 in File S1).

### Data normalization and visualization

Numbers of mapped reads were normalized by the size of each reference genome in order to obtain the relative abundance of each reference genome in each sample. Therefore, the normalized mapped reads ratio for each reference genome of any given sample was calculated by using (mapped read count/reference genome size)/(total mapped read count/total reference genome size). The reference genomes per genus in each body site that have >0.0001 (>0.01%) of the normalized mapped reads from all 16 body sites were retained as the core human microbiome. All reference genomes per genus in each body site that reached >0.025 (2.5%) of the normalized mapped reads from at least one sample were retained for comparative and clustering analysis of community composition.

### Clustering analysis of community composition

Two sets of data, 14 samples from 7 body sites of the same individual and 41 samples from 12 body sites of different individuals, were clustered by R with the ade4 package using Unweighted Pair Group Method with Arithmetic Mean clustering. A singular exception is the Posterior fornix site where only one sample was used (SRS011269) due to discrepancies in sample SRS011111; further explained in the discussion. The number of reads mapped to dominant genera found in 34 samples (16 body sites of different individuals) were imported into the Qiime package [17] for beta diversity analysis using Bray Curtis metrics, and visualized with KiNG (http://kinemage.biochem.duke.edu/software/king.php). Abundance measures of 594 genera from each site were used as input and each sub site was used as the instrumental variable (qualitative factor).

## Results

### Aligning shotgun metagenomes to reference genomes

A total of 55 metagenomic samples from 5 major body sites (including 16 sub-sites) from 14 healthy individuals were selected for this study, including samples from airways (anterior nares), skin (retroauricular crease), oral cavity, gastrointestinal tract (stool), and urogenital tract/vagina (Table S1 in File S2). Although all these samples were sequenced using the Illumina GAIIx platform with 2 lanes of 101 bp paired-end reads per sample, after filtering for both human contamination and quality, an average of 38M reads per sample (ranging from 0.5M to 238M) were available for analysis. These remaining high quality 197 Gbp from all 55 samples were used for read-mapping analysis.

All shotgun reads from 55 metagenomes were aligned to 2780 available reference genomes, in order to assess genome coverage and abundance (Table S1 in File S2). In most cases, a significant fraction (average of 62.3%) of the reads was aligned with the reference genomes. Among the metagenomes studied, reads from the urogenital tract/vagina (posterior fornix)(SRS011111, SRS011269) displayed the highest mapping percentages at 91.6% and 88.1%, respectively, while the skin (retroauricular crease) (SRS016944, SRS015381) displayed the lowest at 20.2% and 12.8%, respectively), largely due to the higher diversity of this open environment [18], [19], [20] (Table S1 in File S2).

### Site-specific references are key

Given the wide range of read mapping results, we wanted to investigate the relative contribution of the reference genomes to see if a rational approach to reference genome sequencing could contribute to greater understanding of metagenomic data derived from samples of the same site. We therefore considered samples taken from 5 major body sites (oral, skin, airway, gastrointestinal tract, and urogenital tract/vagina) and the relative contribution of references derived from those sites. For each metagenome, we divided all 2780 references into three categories (see Table S2 in File S2): 1) those affiliated with the same body site as the metagenome (ranging from 119–424 references); 2) those affiliated with other human body sites (ranging from 1162–1464 references); 3) those not affiliated with the human body (1197 references).

Despite the much larger number of references, the contribution of non-human associated reference genomes was minimal for all samples (average of 0.97%) (Table S3 in File S2). On the other hand, reference genomes associated with the human body (both specific sites and other human body sites) (1583 of 2780 total references) allowed the characterization of 99.03% (140 million) of all mapped reads. Furthermore, among all reads that were mapped to the reference genomes, 75.38% of the human microbiome shotgun metagenomes could be mapped to body site-specific references (Table S3 in File S2), however the relative contribution of references specific to the body site varied depending on the major body site sampled (Figure 1 and Figure S1 in File S1, Table S1 in File S2 and Table S3 in File S2). While some microbiome samples displayed very high read mapping to body site-specific references (up to >99% or 75M reads), others did not map as well (as low as 6%) (Table S3 in File S2), indicating that additional references for those sites would be helpful. This is not surprising in that some body sites are selective and possibly more stable, while others, such as the oral cavity and airway sites, are open systems with dynamic communities, where exogenous microorganisms from the environment are continuously introduced by breathing, eating, and drinking [4].

Approximately 90% of the mapped reads from all urogenital tract and vagina samples could be aligned to the 143 reference genomes (only 5% of the references) associated with the urogenital tract (Table S2 in File S2). In contrast, despite having three times more site-specific references (424, or 15% of all references), less than 60% of the gastrointestinal tract metagenome reads could be mapped to them (Figure 1 in File S1). This highlights the diversity as well as the need for additional reference genomes for the gastrointestinal tract to allow more complete analysis. Similarly, additional site-specific references could benefit the airway, skin, and oral body sites, which also display 60–70% read mapping to reference genomes. A number of references isolated from different body sites did contribute to the characterization of approximately 15–20% of the airway and oral sites, respectively. Therefore additional references isolated from anywhere on the human body may have unexpected contributions to understanding other body site microbiomes. This may be due to similar taxa inhabiting multiple body sites, or alternatively, errors in metadata associated with samples, or sampling issues. Additionally, among samples taken from the same sub-site, the relative contribution of site-specific references, other human body isolates, and references not associated with humans, suggests a highly variable microbiome for some sub-sites, and argues for the acquisition of additional references to better understand the human microbiome.

**Figure 2:**
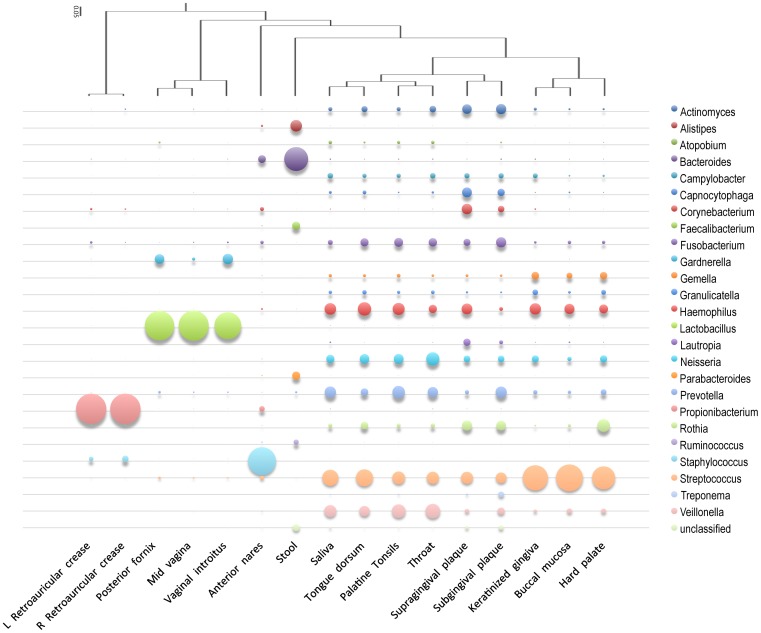
Hierarchical clustering of genus-level microbiome community composition of 16 sampled human body sites. Genus-level abundances were examined for genera whose references accrued >0.025 (2.5%) of the mapped reads from at least one sample. The cumulative genus-level (Y-axis) mapped reads from different body/sub-sites (X-axis) are represented by the size of the bubbles. The samples were clustered based on community composition (tree on top of graph).

Each body site displayed different community profiles, as shown at the genus level in Figure 2 and Table S4 in File S2. While some site-specific references were highly represented in some datasets, other reference genomes were not representative of samples even if they were isolated from the same site. Environments that display poor mapping to references are thus most likely due to higher alpha or beta diversity, coupled with a lack of site-specific references. Although perhaps not possible for all environments, future efforts for reference genome sequencing should try to cover the breadth and depth of the microbial diversity for the site in question.

### Spatial and temporal effects on microbial diversity

Read mapping also allowed us to ask whether there are core organisms (i.e. genomes) present in all body-sites or sub-sites. Not a single reference genome was universally found above 0.01% of mapped reads in all body site samples. Furthermore, we pooled the samples from all 14 individuals and all 16 sampling locations, and grouped the reference genomes at the genus and family levels. Even at these higher levels of taxonomy, only 3 of 595 genera (*Bacteroides, Prevotella, and Streptococcus*) and 5 of 234 families (*Bacteridaceae, Clostridiaceae, Clostridiales, Prevotellaceae, Streptococcaceae*) appear to be universally present in every human body at the same 0.01% threshold, supporting the results of some recent studies showing a minimal membership core [7], [21]. A larger number of taxa are shared among the sub-sites within a major body site. For example, 114 references, 31 genera, and 27 families are shared among 9 oral sub-sites from our pooled samples (Table S5 in File S2), which is consistent with recent rRNA gene based observations of large overlap among oral microbiome samples [22], [23].

**Figure 3:**
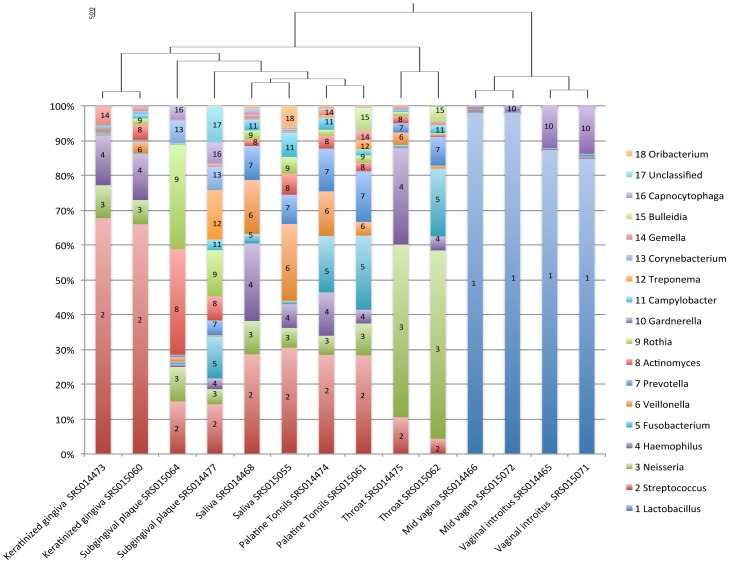
Spatial and temporal analysis of microbial communities within a single individual. The proportion of genus-level assignments of reads is presented from two time-points for each of 7 sites within subject 763577454. Clustering of the samples based on microbiome composition shows conserved community structure of the same site over time, with a singular exception of the two subgingival plaque samples. Results are displayed for genera whose references accrued >0.05 (5%) of the mapped reads from at least one sample.

We selected to examine the abundant human microbiome members at both the genus (Figure 2 and Figure S2 in File S1 and Table S5 in File S3) and family (Table S5 in File S3) levels using a read-mapping cutoff of >2.5% of the metagenomic reads from at least one of the 55 datasets. We examined the correlation of communities between different body sites and found that the 16 sub-sites do cluster in distinct groupings, regardless if they are pooled from many individuals (Figure 2) or if they are from a single individual at different time points (Figure 3). Interestingly, the microbiomes of the oral, gastrointestinal and nasal cavities cluster together, possibly suggesting similar seeding and subsequent colonization of these open environments (Figure 2).

Despite the gross similarity between nearby sub-sites, even when looking at the pooled microbiome of several individuals (Figure 2), there are some notable sub-site differences even within an individual. For example, although the microbiomes of keratinized gingiva and subgingival plaque are separated by only millimeters in distance, they exhibit different community structures within the single individual (Figure 3). Despite mostly clustering together, samples collected from the same sub-site at different time points within the same individual did display some community composition differences, as seen in the subgingival plaque, keratinized gingiva, palatine tonsils, and throat samples. These observations support the results of some recent studies showing that the species-level ‘core community’ at any sub-site is small, with large variations in species abundance [7], [24]. Our results also bolster recent 16S rRNA gene based time-course observations [8], which suggest pronounced variability in an individual's microbiota across months, weeks and even days. Despite these community variations over time, our analyses suggest that the human body's different microenvironments have a stronger influence on microbial community structure. This is evidenced by clustering analysis, where variation within a site over time is smaller than the variation observed among sub-sites (Figure 3).

### Microbiome variation among humans

**Figure 4:**
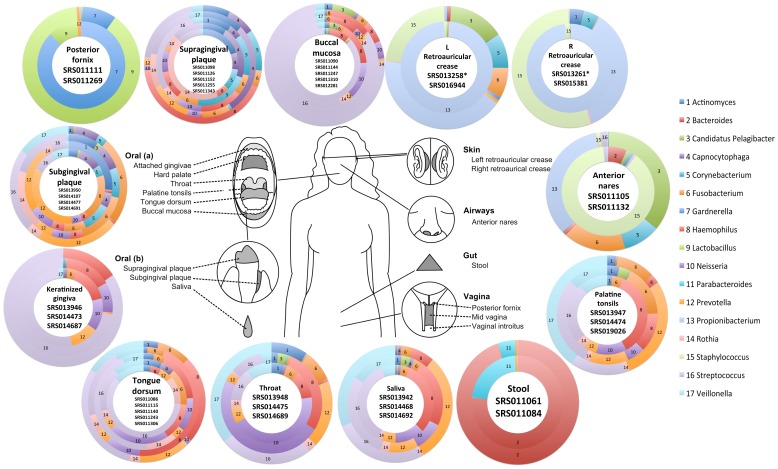
Genus-level comparisons of 13 body site microbiomes among two to five different subjects. The proportion of genus-level assignments of reads is presented as differentially colored slices of doughnut charts per body site. The results from different subjects are presented from the inner to outer rings of the doughnuts. Results are displayed for genera whose references accrued >0.15 (15%) of the mapped reads from at least one sample.

Despite the relative consistency observed in sub-site microbiomes within an individual, we wanted to address the question of person-to-person sub-site microbiome variability. We utilized the read mapping results to compare the presence and abundance of major genera among two or more individuals sampled at the same sub-site (Figure 4). While some sub-sites such as stool show genera abundance consistency among two or more samples, others appear to have higher inter-person variation (Figure 4). Many of these sites such as the anterior nares and retroauricular crease samples are sites that are continuously exposed to circulating non-indigenous organisms that may only have a transient presence in these environments. While left and right retroauricular crease samples from the same individual confirmed previously reported skin habitat body symmetry [6], samples from different individuals at either sub-site differed (Figure 4, Figure S3 in File S1).

Interestingly, this inter-person variability was also observed in less complex and more closed environments, such as in the urogenital tract that is typically dominated by *Lactobacillus* in healthy individuals. Surprisingly, one sample (posterior fornix, SRS011111) has a microbiome dominated by *Gardnerella vaginalis* (Figure S4 in File S1), a facultative anaerobic gram-variable rod that can cause bacterial vaginosis in some women. Although *G. vaginalis* is a major species present in bacterial vaginosis, it has also been isolated from women without any signs or symptoms of infection [25]. It is unclear if this individual was a healthy carrier of *G. vaginalis* who would always remain asymptomatic, or was simply asymptomatic at the time of sampling; which highlights the importance of rigorously defined attributes for sample metadata. In this case, all samples were thought to have been derived from healthy individuals, and it will be of increasing importance to establish appropriate subject recruitment and clinical sampling criteria to avoid potential pitfalls in any downstream analyses. In addition, since we do not yet fully understand what constitutes a healthy or, more generally, a “normal microbiome”, a common practice of analyzing pooled samples from a group of “healthy” individuals may mask the diversity within the population.

**Figure 5:**
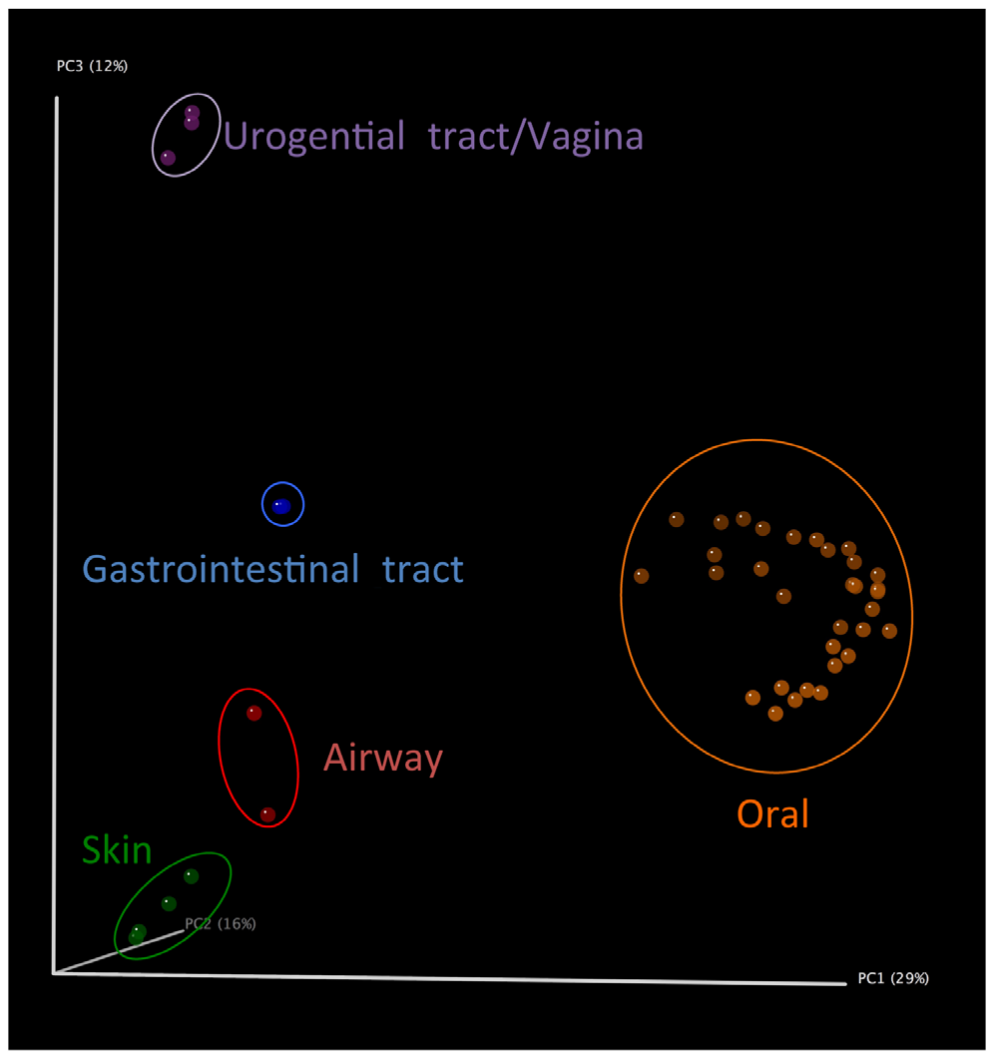
Principal coordinate analysis (PCoA) of the communities from 5 body sites, including 16 sub sites. PCoA clustering is based on read-mapping-based genus-level composition of 43 shotgun metagenomes. Each point corresponds to a sample colored by major body habitat.

Clustering of the same genus-level composition data distinguishes among the five major body sites (Figure 5). However, within the large oral cavity cluster, we only observe loose groupings of samples by sub-site (e.g. Buccal mucosa in Figure S5A in File S1). Groupings of sub-sites did conform to previous 16S rRNA gene based studies, with Buccal mucosa, Hard palate, and Keratinized gingiva in one group (Group I); Palatine tonsils, Saliva, Throat, and Tongue dorsum in Group II; Subgingival and Supragingival plaque in Group III; and Stool as Group 4 (Figure S5B in File S1). These results adhere to the idea that some selective pressure for particular community membership may be exerted on distinct niche environments due to possible interactions with host cell type, saliva chemistry, and other exogenous factors (such as oxygen availability and nutrient deposition) [26]. Despite these relatively robust groupings however, the effects of the individual sampled could be observed in some cases in the oral environment (E.g. sub-sites for subjects 763961826, 763496533, and 158499257; highlighted with arrows in Figure S5 in File S1). Within an environment that may experience frequent mixing, such as the oral cavity, perhaps it is not surprising that we see a subject-specific effect on the microbial communities. A better understanding of what contributes to shaping the human microbiome will require deeper and broader microbiome studies over the human life span, and across people of different genders, ethnicities, lifestyles and cultural habits, geographic locales, and other environmental factors, as these may all contribute to the diversity and variations we are only now becoming familiar with in the human microbiome [27], [28], [29].

## Discussion

The human microbiome comprises diverse and dynamic communities whose role in human health and disease has yet to be fully uncovered. Here, we show the utility of studying human microbiome datasets using reference genomes, and argue in favor of habitat-specific sampling to increase our reference genome repertoire and increase the value of this type of analysis. Using a collection of 55 microbiome samples from 5 sites (and 16 sub-sites) within 14 human individuals, we show that major body sites display persistent groups of microbes among healthy members of the human population. Despite gross similarities however, it is clear that the human microbiome varies by body site, as well as by individual when studying identical sites, and that small spatial as well as temporal variations can exist even within individuals. Although much of this diversity remains unexplained, factors such as diet, environment, host genetics, and early microbial establishment might all be implicated [12]. Such fluctuations in the microbiome of individuals over time will need to be taken into account when comparing individuals to one another.

Another aspect of diversity includes population or strain-level variation, which is only now being investigated in these datasets. From within any given sample, it is expected that variation will exist even within a species in the form of single nucleotide polymorphisms, insertion/deletions, and rearrangements. Such additional information could shed insight into clonal blooms and selective pressure, from either the host or other members of the community, on a species/population.

Despite using a relatively limited, 2780 reference genome set available for this study, we show that an average of 62.3% of reads are sufficiently similar to one of the reference genomes to be assigned confidently. This compares very favorably to the first dental plaque metagenome study, where only 4% of the reads could be mapped to 50 available oral reference genomes [30], demonstrating the utility of having additional reference genomes. Furthermore, given that on average, 58.8% of all mapped metagenome data correspond to organisms isolated from the same body site as the metagenome sample, this argues for site-specific reference genome sampling. Given the observed microbial community differences from person to person and even temporal dynamics within a single individual, it appears that it is important to obtain reference genomes from the same sample undergoing metagenomic sequencing. This may be of even greater importance when studying other, more complex systems.

In this study, as little as 13% of the reads from a skin sample could be mapped onto the available reference genomes, arguing for additional reference sequences. While the HMP consortium has already generated genomes for many of the most abundant human microbiome organisms, there exist efforts to continue to sequence reference genomes targeting specific taxa such as the HMP most wanted list (e.g. http://hmpdacc.org/most wanted/) for observed 16S sequences whose organisms/genomes have thus far proven difficult to isolate/obtain. For those taxa that are found in relatively high abundance and with low intraspecies genomic variation, *de novo* metagenome assembly methods may allow reconstruction of nearly complete genomes [31], [32], [33], [34], which can then be used as reference genomes for the sampled sites. Regardless the method employed, continued sampling and sequencing efforts will undoubtedly need to be performed if we are to truly cover the breadth and depth of human-associated microbial diversity.

## Supporting Information

File S1 Figure S1 in File S1 and in File S4: Metagenome read mapping of pooled samples from five major body sites against three categories of reference genomes (those found in the same body site, a different body site, or not human affiliated). Figure S2 in File S1: Genus-level community composition of the human microbiome per body site. Figure S3 in File S1: Genus-level community composition of the retroauricular crease microbiome. Figure S4 in File S1: Genus-level community composition of the urogenital tract/vagina microbiome. Figure S5 in File S1: Principal coordinate analysis (PCoA) of oral sub-sites and of stool.
(DOCX)

File S2 Table S1 in File S2: Summary of 55 HMP samples and reference genome mapping data. Table S2 in File S2: Number of reference genomes for each of three categories (from the same body site, from a different body site, and not human affiliated), and based on the major body site sampled. Table S3 in File S2: Percentage of all mapped reads corresponding to three reference genome categories: not human affiliated, found in a different body site, and found in the same body site). Table S4 in File S2: Summary of 16 body sites and related reference genus-level mapping data.
(DOCX)

File S3 Table S5 in File S3: Community composition of (A) genus and (B) family -level read mapping assignments.
(XLSX)

File S4 Figure S1 in File S1 and in File S4: Metagenome read mapping of pooled samples from five major body sites against three categories of reference genomes (those found in the same body site, a different body site, or not human affiliated).
(TIF)
