# Supplementary material for: Recruiting Human Microbiome Shotgun Data to Site-Specific Reference Genomes
Source: PLoS One. 2014 Jan 15;9(1):e84963. doi: 10.1371/journal.pone.0084963 (PMC3893169; doi:10.1371/journal.pone.0084963)
Supplement: File S1 — Figure S1 in File S1 and in File S4: Metagenome read mapping of pooled samples from five major body sites against three categories of reference genomes (those found in the same body site, a different body site, or not human affiliated). Figure S2 in File S1: Genus-level community composition of the human microbiome per body site. Figure S3 in File S1: Genus-level community composition of the retroauricular crease microbiome. Figure S4 in File S1: Genus-level community composition of the urogenital tract/vagina microbiome. Figure S5 in File S1: Principal coordinate analysis (PCoA) of oral sub-sites and of stool. (DOCX) [file pone.0084963.s001.docx]

**Supporting Material for**

**Recruiting human microbiome shotgun data to site-specific reference genomes**

**Gary Xie^1,2^, Chien-Chi Lo^1,2^, Matthew Scholz^1,2^, Patrick S. G. Chain^1,2^***

**^1^ Genome Science Group, Los Alamos National Laboratory, Los Alamos, NM 87545;**

**^2^ Microbial and Metagenome Program, Joint Genome Institute, Walnut Creek, CA 94598**

**Supplemental Figures S1-S5**

**Supplemental Figures**





Figure S1: Metagenome read mapping of pooled samples from five major body sites against three categories of reference genomes (those found in the same body site, a different body site, or not human affiliated). The percentage of reads mapped to each reference is shown along the y-axis. Reference genomes are sorted from the highest to lowest in terms of percentage of reads mapped within each of the three categories (shown along the x-axis). The dominant six references from the body site specific category are labeled in green. A few key references from the ‘different body site’ category are labeled in red for the airway, skin and oral body sites. In a singular case (airway sample SRS011132), a reference from the ‘not human affiliated’ category, *Candidatus Pelagibacter ubique SAR11 HTCC1002*, was found to have an unusually high number of mapped reads (in blue). Further analysis shows that only 0.04% of the reference genome was mapped to by ~6% of the combined metagenome reads, and this is solely due to human sequence present in both the reference sequence and this particular metagenome sample, highlighting the need for i) curation of databases from “obvious” contamination; ii) removal of host data from host-derived microbiome samples; iii) rigorous review of ‘big data’ analysis results, which if left unchecked, could be potentially misleading.

Figure S2: Genus-level community composition of the human microbiome per body site. Genus-level abundances were examined for genera whose reference genomes accrued >0.025 (2.5%) of the mapped reads from at least one sample. Various genus are represented on the *y*‐axis and different human body/sub-sites are listed on the *x*‐axis. The ratio of genus-level mapped reads **from 14 individuals are represented by the height of the columns** along the z-axis.


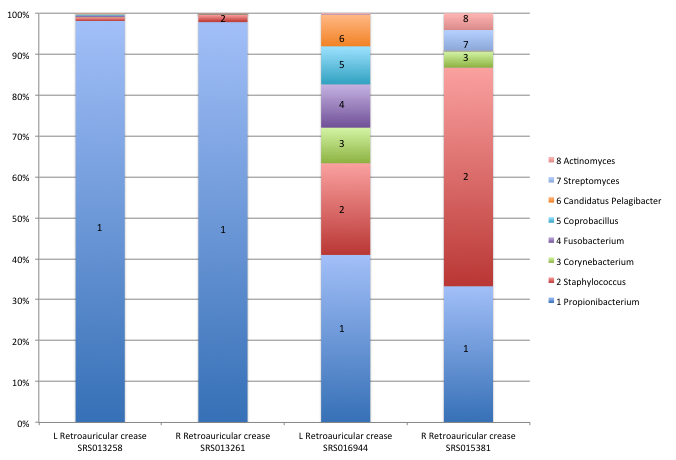


Figure S3: Genus-level community composition of the retroauricular crease microbiome. Genus-level abundances were examined for genera whose reference genomes accrued >0.025 (2.5%) of the normalized mapped reads from at least one of four samples. Proportions of genus level read mapping assignments (Y-axis) **from** retroauricular crease samples from three **individuals** (X-axis) **are represented by the height of the color-coded histogram.** The retroauricular crease samples from 3 individuals are in lane 1-4 (subject 159591683,159591683, 159450072, and 764042746) respectively.
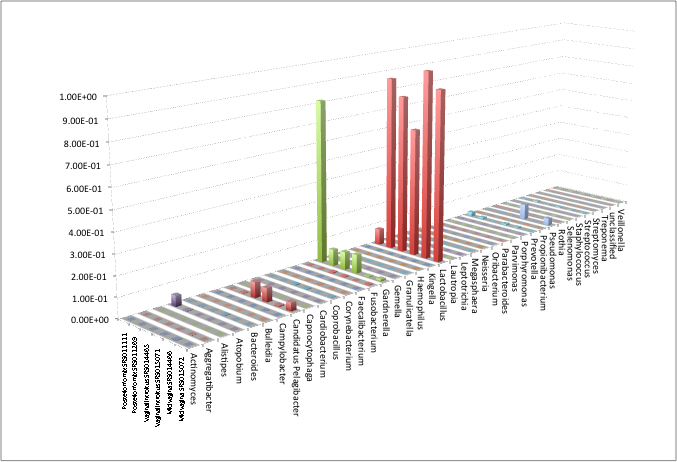


Figure S4: Genus-level community composition of the urogenital tract/vagina microbiome. Genus-level abundances were examined for genera whose reference genomes accrued >0.025 (2.5%) of the normalized mapped reads from at least one sample. Various genus are represented on the *y*‐axis and 6 samples from 3 different urogenital tract/vagina sites are listed on the x‐axis. The ratio of genus-level mapped reads **are represented by the height of the columns** along the z-axis. Posterior fornix (SRS011111 & SRS011269), vaginal introituses (SRS015071 & SRS014465), and mid vagina (SRS014466 & SRS015072) samples are in lane 1-2, 3-4, and 5-6 respectively.


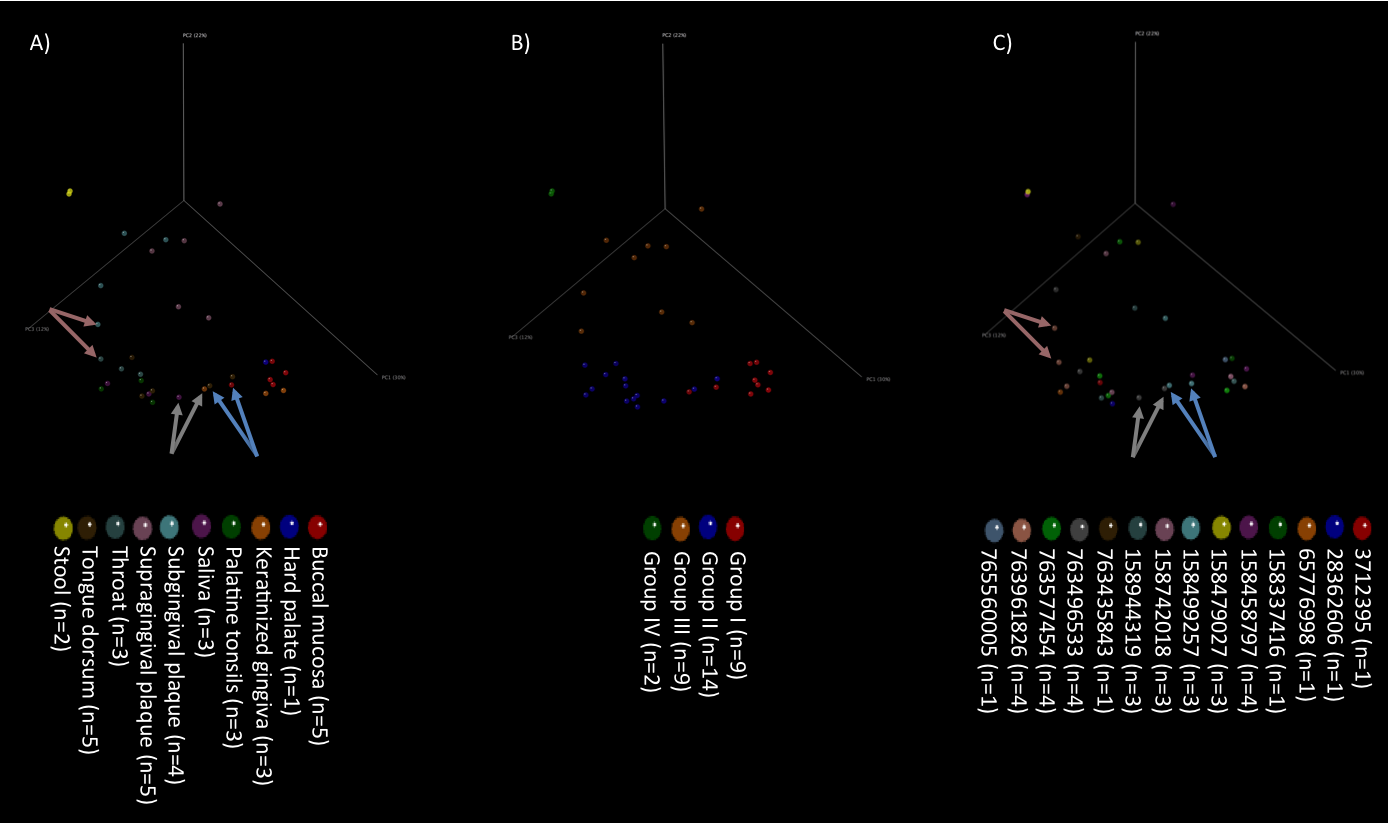


Figure S5: Principal coordinate analysis (PCoA) of oral sub-sites and of stool. Ten sub-sites from 11 individuals (34 total samples) were analyzed and categorized either based on the sub-sites sampled (A), based on one of four previously defined groupings of sub-sites (B), or based on the subject sampled (C). In B, the four groupings consist of Buccal mucosa, Hard palate, and Keratinized gingiva (Group I); Palatine tonsils, Saliva, Throat, and Tongue dorsum (Group II); Subgingival plaque and Supragingival plaque (Group III); and stool (Group IV) as the outgroup. Some loose clustering of metagenomes from the same individual are highlighted with arrows; three individuals, subjects 763961826 (pink), 763496533(grey), and 158499257 (blue).
