# Supplementary material for: Recruiting Human Microbiome Shotgun Data to Site-Specific Reference Genomes
Source: PLoS One. 2014 Jan 15;9(1):e84963. doi: 10.1371/journal.pone.0084963 (PMC3893169; doi:10.1371/journal.pone.0084963)
Supplement: File S2 — Table S1 in File S2: Summary of 55 HMP samples and reference genome mapping data. Table S2 in File S2: Number of reference genomes for each of three categories (from the same body site, from a different body site, and not human affiliated), and based on the major body site sampled. Table S3 in File S2: Percentage of all mapped reads corresponding to three reference genome categories: not human affiliated, found in a different body site, and found in the same body site). Table S4 in File S2: Summary of 16 body sites and related reference genus-level mapping data. (DOCX) [file pone.0084963.s002.docx]

**Supporting Material for**

**Recruiting human microbiome shotgun data to site-specific reference genomes**

**Gary Xie^1,2^, Chien-Chi Lo^1,2^, Matthew Scholz^1,2^, Patrick S. G. Chain^1,2^***

**^1^ Genome Science Group, Los Alamos National Laboratory, Los Alamos, NM 87545;**

**^2^ Microbial and Metagenome Program, Joint Genome Institute, Walnut Creek, CA 94598**

**Supplemental Tables S1-S4**

Table S1: Summary of 55 HMP samples and reference genome mapping data.

| Sample ID^#^ | Subject ID | # of reads after QC and de-contamination | Total bp of reads after QC and de-contamination | # of reads mapped to reference | % of reads mapped to references | Reference genome with the highest average mapping coverage in fold | Highest fold of average mapping coverage | Reference genome with the highest % mapping coverage) | Highest % of mapping coverage | Reference genome with the highest mapped reads ratio^ | Highest normalized mapped reads ratio* |
| --- | --- | --- | --- | --- | --- | --- | --- | --- | --- | --- | --- |
| Oral |  |  |  |  |  |  |  |  |  |  |  |
| Keratinized gingiva |  |  |  |  |  |  |  |  |  |  |  |
| SRS013946 | 763496533 | 76400632 | 7639545128 | 32072949 | 42.0% | Campylobacter concisus 13826 | 162.93 | Prevotella sp. F0039 | 89.47 | Campylobacter concisus 13826 | 10.94 |
| SRS014473 | 763577454 | 56399156 | 5529969188 | 40030293 | 71.0% | Streptococcus mitis SK321 | 292.98 | Streptococcus mitis SK321 | 92.8 | Streptococcus mitis SK321 | 14.02 |
| SRS014687 | 763961826 | 99087632 | 9762005285 | 73828901 | 74.5% | Streptococcus mitis NCTC 12261 | 743.9 | Streptococcus mitis NCTC 12261 | 96.82 | Streptococcus mitis NCTC 12261 | 18.93 |
| SRS015060 | 763577454 | 37237621 | 3519253669 | 26360166 | 70.8% | Streptococcus mitis SK321 | 154.28 | Actinomyces viscosus C505 | 94.89 | Streptococcus mitis SK321 | 11.37 |
| SRS019025 | 763961826 | 8059123 | 767819886 | 6053324 | 75.1% | Streptococcus mitis NCTC 12261 | 57.8 | Streptococcus mitis NCTC 12261 | 96.41 | Streptococcus mitis NCTC 12261 | 18.20 |
| Buccal mucosa |  |  |  |  |  |  |  |  |  |  |  |
| SRS011090 | 158458797 | 3857363 | 368116308 | 2955368 | 76.6% | Streptococcus mitis NCTC 12261 | 21.76 | Streptococcus mitis NCTC 12261 | 86.48 | Streptococcus mitis NCTC 12261 | 14.04 |
| SRS011144 | 158499257 | 3602113 | 304761048 | 2222532 | 61.7% | Streptococcus mitis NCTC 12261 | 4.38 | Gemella haemolysans ATCC 10379 | 78.77 | Neisseria sicca ATCC 29256 | 6.49 |
| SRS011247 | 158742018 | 25027018 | 2250435608 | 18987767 | 75.9% | Streptococcus mitis SK564 | 84.56 | Streptococcus mitis NCTC 12261 | 94 | Streptococcus mitis SK564 | 10.04 |
| SRS011310 | 158944319 | 21006750 | 1935057053 | 15884790 | 75.6% | Streptococcus mitis SK564 | 72.89 | Streptococcus mitis NCTC 12261 | 92.98 | Streptococcus mitis SK564 | 10.14 |
| SRS012281 | 158337416 | 1260616 | 119134388 | 816874 | 64.8% | Streptococcus mitis SK564 | 3.99 | Haemophilus parainfluenzae T3T1 | 68.91 | Streptococcus mitis SK564 | 10.20 |
| Hard palate |  |  |  |  |  |  |  |  |  |  |  |
| SRS062878 | 765560005 | 59171049 | 5708267888 | 45883700 | 77.5% | Streptococcus mitis NCTC 12261 | 198.5 | Gemella haemolysans ATCC 10379 | 96.36 | Rothia mucilaginosa ATCC 25296 | 8.86 |
| Palatine Tonsils |  |  |  |  |  |  |  |  |  |  |  |
| SRS013947 | 763496533 | 28362606 | 2836256835 | 18396288 | 64.9% | Haemophilus parainfluenzae T3T1 | 257.03 | Haemophilus parainfluenzae T3T1 | 94.64 | Haemophilus parainfluenzae T3T1 | 21.60 |
| SRS014474 | 763577454 | 3712395 | 350466113 | 1916483 | 51.6% | Haemophilus parainfluenzae T3T1 | 7.14 | Haemophilus parainfluenzae T3T1 | 86.31 | Haemophilus parainfluenzae T3T1 | 15.96 |
| SRS015061 | 763577454 | 4010587 | 383417323 | 1744832 | 43.5% | Bulleidia extructa W1219 | 4.22 | Bulleidia extructa W1219 | 91.09 | Prevotella oris C735 | 13.04 |
| SRS019026 | 763961826 | 27963070 | 2709675017 | 13933411 | 49.8% | Veillonella dispar ATCC 17748 | 78.05 | Veillonella dispar ATCC 17748 | 97.48 | Veillonella dispar ATCC 17748 | 5.83 |
| SRS019126 | 763496533 | 65776998 | 6263370919 | 45514315 | 69.2% | Veillonella sp. oral taxon 158 F0412 | 207.5 | Veillonella sp. oral taxon 158 F0412 | 95.9 | Prevotella melaninogenica ATCC 25845 | 11.72 |
| Saliva |  |  |  |  |  |  |  |  |  |  |  |
| SRS013942 | 763496533 | 8279284 | 769095580 | 5377987 | 65.0% | Haemophilus parainfluenzae T3T1 | 52.31 | Haemophilus parainfluenzae T3T1 | 94.24 | Haemophilus parainfluenzae T3T1 | 14.03 |
| SRS014468 | 763577454 | 2464625 | 233112682 | 1298932 | 52.7% | Haemophilus parainfluenzae T3T1 | 9.47 | Haemophilus parainfluenzae T3T1 | 91.11 | Haemophilus parainfluenzae T3T1 | 8.41 |
| SRS014692 | 763961826 | 14637415 | 1414212064 | 7797340 | 53.3% | Veillonella dispar ATCC 17748 | 36.66 | Veillonella dispar ATCC 17748 | 97.6 | Prevotella melaninogenica ATCC 25845 | 6.94 |
| SRS015055 | 763577454 | 8808132 | 859574626 | 4565896 | 51.8% | Veillonella dispar ATCC 17748 | 11.86 | Oribacterium sinus F0268 | 93.83 | Oribacterium sinus F0268 | 3.02 |
| SRS019120 | 763496533 | 8198404 | 783553313 | 4945026 | 60.3% | Haemophilus parainfluenzae T3T1 | 26.82 | Haemophilus parainfluenzae T3T1 | 94.52 | Haemophilus parainfluenzae T3T1 | 7.07 |
| Subgingival plaque |  |  |  |  |  |  |  |  |  |  |  |
| SRS013950 | 763496533 | 28293689 | 2662306479 | 14367659 | 50.8% | Prevotella tannerae ATCC 51259 | 50.07 | Capnocytophaga ochracea VPI 2845, DSM 7271 | 94.41 | Prevotella sp. F0108 | 11.32 |
| SRS014107 | 763435843 | 11784578 | 1012188241 | 3902740 | 33.1% | Prevotella tannerae ATCC 51259 | 9.97 | Bacteroidetes sp. F0058 | 87.99 | Prevotella tannerae ATCC 51259 | 7.01 |
| SRS014477 | 763577454 | 11592657 | 1073769961 | 4941138 | 42.6% | Treponema vincentii ATCC 35580 | 18.57 | Rothia dentocariosa M567 | 96.15 | Treponema vincentii ATCC 35580 | 10.43 |
| SRS014691 | 763961826 | 27845487 | 2648624190 | 14607415 | 52.5% | Prevotella tannerae ATCC 51259 | 36.72 | Bulleidia extructa W1219 | 95.61 | Prevotella tannerae ATCC 51259 | 6.98 |
| SRS015064 | 763577454 | 24971889 | 2224753017 | 15603449 | 62.5% | Rothia dentocariosa M567 | 74.09 | Actinomyces sp. oral taxon 171 F0337 | 98.14 | Rothia dentocariosa M567 | 13.11 |
| Supragingival plaque |  |  |  |  |  |  |  |  |  |  |  |
| SRS011098 | 158458797 | 56414058 | 4847043481 | 36094138 | 64.0% | Rothia dentocariosa ATCC 17931 | 231.72 | Kingella oralis UB-38, ATCC 51147 | 98.67 | Rothia dentocariosa M567 | 18.84 |
| SRS011126 | 158479027 | 108042209 | 9974154224 | 64751856 | 59.9% | Lautropia mirabilis ATCC 51599 | 251.62 | Leptotrichia goodfellowii F0264 | 99.65 | Lautropia mirabilis ATCC 51599 | 14.67 |
| SRS011152 | 158499257 | 69319249 | 6441626905 | 46624467 | 67.3% | Haemophilus parainfluenzae T3T1 | 207.77 | Actinomyces viscosus C505 | 98.64 | Haemophilus parainfluenzae T3T1 | 9.78 |
| SRS011255 | 158742018 | 40060319 | 3581582392 | 20484020 | 51.1% | Capnocytophaga sputigena Capno, ATCC 33612 | 50.77 | Actinomyces sp. oral taxon 171 F0337 | 96.25 | Capnocytophaga sputigena Capno, ATCC 33612 | 7.81 |
| SRS011343 | 158944319 | 45117680 | 3973590492 | 27828278 | 61.7% | Haemophilus parainfluenzae T3T1 | 334.28 | Streptococcus sanguinis SK36 | 96.4 | Haemophilus parainfluenzae T3T1 | 27.50 |
| Throat |  |  |  |  |  |  |  | oral |  |  |  |
| SRS013948 | 763496533 | 31107576 | 2942725911 | 20906453 | 67.2% | Neisseria subflava NJ9703 | 112.41 | Veillonella sp. oral taxon 158 F0412 | 93.85 | Neisseria subflava NJ9703 | 13.03 |
| SRS014475 | 763577454 | 1485019 | 138112413 | 1047800 | 70.6% | Neisseria meningitidis MC58 | 3.71 | Neisseria meningitidis MC58 | 62.23 | Neisseria meningitidis MC58 | 8.72 |
| SRS014689 | 763961826 | 21825317 | 2055696315 | 13496638 | 61.8% | Veillonella dispar ATCC 17748 | 72.89 | Veillonella dispar ATCC 17748 | 97.09 | Veillonella dispar ATCC 17748 | 12.01 |
| SRS015062 | 763577454 | 1743965 | 169269918 | 1066037 | 61.1% | Neisseria meningitidis MC58 | 3.99 | Bulleidia extructa W1219 | 73.05 | Neisseria meningitidis MC58 | 9.00 |
| SRS019027 | 763961826 | 29030827 | 2786517504 | 16747472 | 57.7% | Veillonella dispar ATCC 17748 | 126.96 | Veillonella dispar ATCC 17748 | 98.09 | Veillonella dispar ATCC 17748 | 16.51 |
| Tongue dorsum |  |  |  |  |  |  |  |  |  |  |  |
| SRS011086 | 158458797 | 81664789 | 7754426553 | 59555933 | 72.9% | Streptococcus salivarius SK126 | 421.46 | Streptococcus parasanguinis F0405 | 96.44 | Streptococcus salivarius SK126 | 15.88 |
| SRS011115 | 158479027 | 166408652 | 14668412888 | 100411423 | 60.3% | Haemophilus parainfluenzae T3T1 | 456.09 | Streptococcus australis ATCC 700641 | 97.8 | Haemophilus parainfluenzae T3T1 | 10.76 |
| SRS011140 | 158499257 | 81772451 | 7875279209 | 55347472 | 67.7% | Haemophilus parainfluenzae T3T1 | 348.54 | Streptococcus australis ATCC 700641 | 98.02 | Haemophilus parainfluenzae T3T1 | 13.72 |
| SRS011243 | 158742018 | 72693775 | 6269622825 | 54796705 | 75.4% | Haemophilus parainfluenzae T3T1 | 871.71 | Veillonella sp. oral taxon 158 F0412 | 96.4 | Haemophilus parainfluenzae T3T1 | 38.60 |
| SRS011306 | 158944319 | 111715428 | 10478415359 | 76517531 | 68.5% | Haemophilus parainfluenzae T3T1 | 633.81 | Oribacterium sinus F0268 | 97.18 | Haemophilus parainfluenzae T3T1 | 18.35 |
|  |  |  |  |  |  |  |  |  |  |  |  |
| Airway |  |  |  |  |  |  |  |  |  |  |  |
| Anterior nares |  |  |  |  |  |  |  |  |  |  |  |
| SRS011105 | 158458797 | 4103788 | 362116371 | 2940494 | 71.7% | Staphylococcus aureus RF122 | 7.4 | Staphylococcus aureus RF122 | 65.06 | Staphylococcus aureus RF122 | 7.25 |
| SRS011132 | 158479027 | 511400 | 45930720 | 205947 | 40.3% | Propionibacterium acnes SK137 | 0.62 | Propionibacterium acnes SK137 | 42.9 | Propionibacterium acnes SK137 | 8.96 |
|  |  |  |  |  |  |  |  |  |  |  |  |
| Skin |  |  |  |  |  |  |  |  |  |  |  |
| L Retroauricular crease |  |  |  |  |  |  |  |  |  |  |  |
| SRS013258 | 159591683 | 23548107 | 2066351936 | 17651046 | 75.0% | Propionibacterium acnes J139 | 153.29 | Propionibacterium acnes J139 | 100 | Propionibacterium acnes J139 | 24.50 |
| SRS016944 | 159450072 | 4970921 | 394696851 | 1005817 | 20.2% | Propionibacterium acnes J165 | 3.16 | Propionibacterium acnes J165 | 92.21 | Propionibacterium acnes J165 | 9.10 |
| R Retroauricular crease |  |  |  |  |  |  |  |  |  |  |  |
| SRS013261 | 159591683 | 55078145 | 4943406747 | 47106510 | 85.5% | Propionibacterium acnes SK187 | 359.72 | Propionibacterium acnes J139 | 100 | Propionibacterium acnes SK187 | 21.70 |
| SRS015381 | 764042746 | 20952929 | 1618398535 | 2681516 | 12.8% | Staphylococcus epidermidis ATCC 12228 | 8.29 | Staphylococcus epidermidis SK135 | 94.86 | Staphylococcus epidermidis ATCC 12228 | 8.30 |
|  |  |  |  |  |  |  |  |  |  |  |  |
| Gastrointestinal tract |  |  |  |  |  |  |  |  |  |  |  |
| Stool |  |  |  |  |  |  |  |  |  |  |  |
| SRS011061 | 158458797 | 90085554 | 8028157196 | 53420789 | 59.3% | Bacteroides cellulosilyticus DSM 14838 | 172.86 | Parabacteroides merdae ATCC 43184 | 92.67 | Bacteroides cellulosilyticus DSM 14838 | 24.13 |
| SRS011084 | 158479027 | 238641707 | 22784512697 | 140295265 | 58.8% | Bacteroides stercoris ATCC 43183 | 595.49 | Akkermansia muciniphila ATCC BAA-835 | 93.12 | Bacteroides stercoris ATCC 43183 | 18.28 |
|  |  |  |  |  |  |  |  |  |  |  |  |
| Urogenital tract/vagina |  |  |  |  |  |  |  |  |  |  |  |
| Mid vagina |  |  |  |  |  |  |  |  |  |  |  |
| SRS014466 | 763577454 | 746982 | 73793919 | 606507 | 81.2% | Lactobacillus crispatus 214-1 | 4.26 | Lactobacillus crispatus 214-1 | 80.33 | Lactobacillus crispatus MV-3A-US | 15.70 |
| SRS015072 | 763577454 | 1067759 | 101933552 | 789382 | 73.9% | Lactobacillus crispatus 214-1 | 4.54 | Lactobacillus crispatus 214-1 | 81.51 | Lactobacillus crispatus MV-3A-US | 13.00 |
| Posterior fornix |  |  |  |  |  |  |  |  |  |  |  |
| SRS011111 | 158458797 | 83056321 | 7890506232 | 76083220 | 91.6% | Gardnerella vaginalis 5-1 | 1522.57 | Gardnerella vaginalis 317, ATCC 14019 | 98.15 | Gardnerella vaginalis 5-1 | 35.30 |
| SRS011269 | 158742018 | 5407699 | 517352638 | 4764261 | 88.1% | Lactobacillus jensenii JV-V16 | 78.36 | Lactobacillus jensenii JV-V16 | 99.92 | Lactobacillus jensenii JV-V16 | 27.10 |
| Vaginal introitus |  |  |  |  |  |  |  |  |  |  |  |
| SRS014465 | 763577454 | 875954 | 85866296 | 539166 | 61.6% | Lactobacillus crispatus 214-1 | 3.18 | Lactobacillus crispatus 214-1 | 77.17 | Lactobacillus crispatus MV-3A-US | 13.20 |
| SRS015071 | 763577454 | 507176 | 47953656 | 343809 | 67.8% | Lactobacillus crispatus 214-1 | 1.2 | Gardnerella vaginalis AMD | 57.79 | Lactobacillus crispatus MV-3A-US | 7.90 |

^#^The sequence data can be retrieved from NCBI Sequence Read Archive (<https://www.ncbi.nlm.nih.gov/sra/>) and DACC HMIWGS/HMASM - Illumina WGS Reads and Assemblies (<http://devel.hmpdacc.org/HMASM/>). ^ In order to avoid misinterpreting spurious matches to genomes, the top reference genomes must have at least 1% genome coverage. * The normalized mapped reads ratio for each reference genome of any given sample was calculated using (mapped read count/reference genome size)/(total mapped read count/total reference genome size).

Table S2: Number of reference genomes for each of three categories (from the same body site, from a different body site, and not human affiliated), and based on the major body site sampled

|  | Not human affiliated | Different body site | Same body site | Total |
| --- | --- | --- | --- | --- |
| Oral | 1197 (43.06%) | 1449(52.12%) | 134(4.82%) | 2780 |
| Airways | 1197 (43.06%) | 1434(51.58%) | 149 (5.35%) | 2780 |
| Skin | 1197 (43.06%) | 1464(52.66%) | 119(4.28%) | 2780 |
| Gastrointestinal tract | 1197 (43.06%) | 1162(41.80%) | 424(15.25%) | 2780 |
| Urogenital tract/vagina | 1197 (43.06%) | 1443(51.91%) | 143(5.14%) | 2780 |

Table S3: Percentage of all mapped reads corresponding to three reference genome categories: not human affiliated, found in a different body site, and found in the same body site).

|  | SRS ID | not human affiliated | different body site | same body site |
| --- | --- | --- | --- | --- |
| Urogenital_tract |  |  |  |  |
| Mid_vagina | SRS014466 | 2.05% | 3.17% | 94.78% |
| Mid_vagina | SRS015072 | 8.67% | 4.02% | 87.31% |
| Posterior_fornix | SRS011111 | 0.07% | 0.88% | 99.04% |
| Posterior_fornix | SRS011269 | 0.55% | 1.73% | 97.72% |
| Vaginal_introitus | SRS014465 | 9.22% | 2.75% | 88.03% |
| Vaginal_introitus | SRS015071 | 16.38% | 7.72% | 75.90% |
| Skin |  |  |  |  |
| L_Retroauricular_crease | SRS013258 | 0.43% | 2.04% | 97.53% |
| L_Retroauricular_crease | SRS016944 | 8.61% | 32.15% | 59.23% |
| R_Retroauricular_crease | SRS013261 | 0.19% | 0.84% | 98.96% |
| R_Retroauricular_crease | SRS015381 | 22.44% | 11.58% | 65.98% |
| Gastrointestinal_tract |  |  |  |  |
| Stool | SRS011061 | 0.96% | 0.68% | 98.36% |
| Stool | SRS011084 | 0.55% | 0.52% | 98.93% |
| Airway |  |  |  |  |
| Anterior_nares | SRS011105 | 0.99% | 18.02% | 80.99% |
| Anterior_nares | SRS011132 | 19.72% | 74.25% | 6.03% |
| Oral |  |  |  |  |
| Buccal_mucosa | SRS011090 | 1.94% | 29.12% | 68.94% |
| Buccal_mucosa | SRS011144 | 3.43% | 43.63% | 52.94% |
| Buccal_mucosa | SRS011247 | 0.90% | 26.71% | 72.39% |
| Buccal_mucosa | SRS011310 | 0.91% | 28.26% | 70.83% |
| Buccal_mucosa | SRS012281 | 4.55% | 28.68% | 66.77% |
| Hard_palate | SRS062878 | 0.52% | 43.17% | 56.31% |
| Keratinized_gingiva | SRS013946 | 0.77% | 36.01% | 63.21% |
| Keratinized_gingiva | SRS014473 | 0.59% | 28.43% | 70.98% |
| Keratinized_gingiva | SRS014687 | 0.39% | 28.62% | 70.99% |
| Keratinized_gingiva | SRS015060 | 0.66% | 31.82% | 67.52% |
| Keratinized_gingiva | SRS019025 | 0.44% | 23.88% | 75.69% |
| Palatine_Tonsils | SRS013947 | 1.12% | 29.15% | 69.72% |
| Palatine_Tonsils | SRS014474 | 2.98% | 40.40% | 56.62% |
| Palatine_Tonsils | SRS015061 | 2.39% | 49.63% | 47.99% |
| Palatine_Tonsils | SRS019026 | 0.68% | 35.46% | 63.85% |
| Palatine_Tonsils | SRS019126 | 0.69% | 30.36% | 68.95% |
| Saliva | SRS013942 | 1.63% | 30.45% | 67.92% |
| Saliva | SRS014468 | 3.03% | 29.27% | 67.70% |
| Saliva | SRS014692 | 0.58% | 29.71% | 69.70% |
| Saliva | SRS015055 | 2.96% | 37.49% | 59.55% |
| Saliva | SRS019120 | 1.36% | 32.67% | 65.97% |
| Subgingival_plaque | SRS013950 | 1.56% | 28.11% | 70.33% |
| Subgingival_plaque | SRS014107 | 2.81% | 29.69% | 67.49% |
| Subgingival_plaque | SRS014477 | 2.14% | 22.27% | 75.59% |
| Subgingival_plaque | SRS014691 | 0.67% | 32.34% | 66.99% |
| Subgingival_plaque | SRS015064 | 1.84% | 28.83% | 69.33% |
| Supragingival_plaque | SRS011098 | 0.83% | 18.74% | 80.44% |
| Supragingival_plaque | SRS011126 | 1.45% | 15.98% | 82.57% |
| Supragingival_plaque | SRS011152 | 0.86% | 28.06% | 71.08% |
| Supragingival_plaque | SRS011255 | 2.12% | 27.44% | 70.44% |
| Supragingival_plaque | SRS011343 | 1.48% | 17.86% | 80.66% |
| Throat | SRS013948 | 0.65% | 45.12% | 54.24% |
| Throat | SRS014475 | 2.86% | 77.86% | 19.28% |
| Throat | SRS014689 | 0.88% | 37.58% | 61.54% |
| Throat | SRS015062 | 2.58% | 75.04% | 22.38% |
| Throat | SRS019027 | 0.60% | 33.05% | 66.35% |
| Tongue_dorsum | SRS011086 | 1.90% | 35.49% | 62.61% |
| Tongue_dorsum | SRS011115 | 0.85% | 43.11% | 56.04% |
| Tongue_dorsum | SRS011140 | 1.11% | 39.28% | 59.62% |
| Tongue_dorsum | SRS011243 | 0.98% | 27.79% | 71.23% |
| Tongue_dorsum | SRS011306 | 1.50% | 28.01% | 70.48% |
| All data: |  | 0.97% | 23.65% | 75.38% |

Table S4: Summary of 16 body sites and related reference genus-level mapping data. Genus-level abundances were examined for genera whose references accrued >0.025 (2.5%) of the mapped reads from at least one sample.

|  | L Retroauricular crease | R Retroauricular crease | Mid vagina | Vaginal introitus | Anterior nares | Posterior fornix | Stool | Keratinized gingiva | Buccal mucosa | Hard palate | Saliva | Tongue dorsum | Palatine Tonsils | Throat | Subgingival plaque | Supragingival plaque |
| --- | --- | --- | --- | --- | --- | --- | --- | --- | --- | --- | --- | --- | --- | --- | --- | --- |
| Actinomyces | 0.03% | 0.17% | 0.07% | 0.01% | 0.05% | 0.00% | 0.01% | 0.86% | 0.39% | 0.43% | 1.55% | 3.57% | 1.58% | 4.25% | 10.33% | 9.14% |
| Alistipes | 0.01% | 0.00% | 0.00% | 0.00% | 0.43% | 0.00% | 13.50% | 0.01% | 0.01% | 0.01% | 0.02% | 0.01% | 0.01% | 0.02% | 0.02% | 0.01% |
| Atopobium | 0.00% | 0.00% | 0.00% | 0.02% | 0.01% | 5.76% | 0.01% | 0.01% | 0.03% | 0.08% | 1.21% | 0.37% | 0.95% | 1.10% | 0.29% | 0.03% |
| Bacteroides | 0.07% | 0.02% | 0.01% | 0.05% | 5.90% | 0.01% | 58.23% | 0.14% | 0.06% | 0.05% | 0.18% | 0.05% | 0.11% | 0.13% | 0.16% | 0.04% |
| Campylobacter | 0.00% | 0.00% | 0.00% | 0.00% | 0.01% | 0.00% | 0.03% | 2.29% | 0.24% | 0.44% | 2.99% | 1.69% | 1.74% | 2.89% | 2.63% | 2.66% |
| Capnocytophaga | 0.01% | 0.00% | 0.03% | 0.00% | 0.01% | 0.00% | 0.00% | 0.10% | 0.29% | 0.08% | 0.86% | 1.38% | 0.21% | 0.57% | 5.16% | 9.67% |
| Corynebacterium | 0.47% | 0.20% | 0.04% | 0.05% | 1.53% | 0.00% | 0.01% | 0.23% | 0.04% | 0.02% | 0.09% | 0.03% | 0.02% | 0.04% | 4.05% | 10.96% |
| Faecalibacterium | 0.00% | 0.00% | 0.00% | 0.00% | 0.11% | 0.00% | 6.47% | 0.00% | 0.01% | 0.00% | 0.01% | 0.01% | 0.01% | 0.01% | 0.01% | 0.00% |
| Fusobacterium | 0.65% | 0.08% | 0.12% | 0.29% | 1.12% | 0.02% | 0.01% | 0.61% | 1.15% | 1.07% | 2.38% | 6.51% | 7.83% | 6.98% | 10.45% | 4.76% |
| Gardnerella | 0.04% | 0.00% | 0.99% | 10.71% | 0.05% | 76.96% | 0.00% | 0.01% | 0.04% | 0.00% | 0.01% | 0.01% | 0.01% | 0.01% | 0.01% | 0.00% |
| Gemella | 0.00% | 0.00% | 0.01% | 0.00% | 0.05% | 0.01% | 0.00% | 5.61% | 3.38% | 5.19% | 1.00% | 0.84% | 1.33% | 0.62% | 0.58% | 0.74% |
| Granulicatella | 0.00% | 0.00% | 0.00% | 0.00% | 0.04% | 0.00% | 0.00% | 3.01% | 0.69% | 2.19% | 1.13% | 1.83% | 1.01% | 1.31% | 0.32% | 0.49% |
| Haemophilus | 0.01% | 0.00% | 0.01% | 0.01% | 0.31% | 0.00% | 0.02% | 12.89% | 10.28% | 7.75% | 13.55% | 18.08% | 14.59% | 6.59% | 1.39% | 11.56% |
| Lactobacillus | 0.02% | 0.01% | 92.21% | 73.18% | 0.03% | 14.16% | 0.01% | 0.03% | 0.08% | 0.04% | 0.10% | 0.05% | 0.03% | 0.03% | 0.03% | 0.02% |
| Lautropia | 0.00% | 0.00% | 0.03% | 0.00% | 0.00% | 0.00% | 0.00% | 0.06% | 0.25% | 0.07% | 0.26% | 0.03% | 0.01% | 0.04% | 1.49% | 5.16% |
| Neisseria | 0.01% | 0.01% | 0.03% | 0.01% | 0.08% | 0.00% | 0.00% | 4.74% | 1.74% | 5.02% | 6.63% | 9.24% | 9.76% | 17.71% | 4.16% | 4.35% |
| Parabacteroides | 0.00% | 0.00% | 0.00% | 0.00% | 0.09% | 0.00% | 6.37% | 0.01% | 0.01% | 0.01% | 0.01% | 0.01% | 0.01% | 0.01% | 0.01% | 0.01% |
| Prevotella | 0.05% | 0.01% | 0.20% | 0.21% | 0.06% | 1.28% | 0.42% | 1.76% | 1.10% | 3.28% | 13.12% | 5.75% | 15.96% | 10.87% | 12.89% | 1.63% |
| Propionibacterium | 93.75% | 93.96% | 0.03% | 0.02% | 2.97% | 0.00% | 0.00% | 0.00% | 0.02% | 0.01% | 0.07% | 0.01% | 0.02% | 0.07% | 0.06% | 0.04% |
| Rothia | 0.02% | 0.02% | 0.03% | 0.01% | 0.09% | 0.00% | 0.00% | 0.31% | 0.98% | 16.31% | 1.64% | 5.69% | 1.61% | 1.87% | 9.50% | 10.16% |
| Ruminococcus | 0.01% | 0.01% | 0.00% | 0.00% | 0.13% | 0.00% | 2.67% | 0.00% | 0.02% | 0.00% | 0.02% | 0.02% | 0.02% | 0.02% | 0.01% | 0.00% |
| Staphylococcus | 1.95% | 3.99% | 0.02% | 0.03% | 79.32% | 0.00% | 0.01% | 0.01% | 0.01% | 0.01% | 0.02% | 0.02% | 0.01% | 0.01% | 0.01% | 0.00% |
| Streptococcus | 0.08% | 0.04% | 0.23% | 0.20% | 1.53% | 0.17% | 0.06% | 64.27% | 73.21% | 53.66% | 26.39% | 29.38% | 16.29% | 15.04% | 11.57% | 14.58% |
| Treponema | 0.00% | 0.00% | 0.00% | 0.00% | 0.00% | 0.00% | 0.00% | 0.00% | 0.00% | 0.01% | 0.13% | 0.04% | 0.22% | 0.02% | 3.40% | 0.38% |
| Veillonella | 0.01% | 0.00% | 0.04% | 0.01% | 0.14% | 0.00% | 0.02% | 1.05% | 2.72% | 2.50% | 14.62% | 10.82% | 18.74% | 21.42% | 5.06% | 1.82% |
| unclassified | 0.01% | 0.01% | 0.01% | 0.04% | 0.09% | 0.11% | 4.83% | 0.02% | 0.04% | 0.02% | 0.44% | 0.08% | 0.14% | 0.10% | 2.04% | 1.02% |

Table S5. Community composition of (A) genus and (B) family -level read mapping assignments. Genus-level abundances were examined for genera whose references accrued >0.025 (2.5%) of the normalized mapped reads from at least one sample.

See additional Supplementary Excel table.
